# Blood Culture-Negative Endocarditis Secondary to Skin Popping

**DOI:** 10.7759/cureus.37617

**Published:** 2023-04-15

**Authors:** Bradley Casey, Abigail Daniels, Alejandro Chapa-Rodriguez, Amol Bahekar, Divyang Patel, Raviteja Guddeti

**Affiliations:** 1 Internal Medicine, Cape Fear Valley Medical Center, Fayetteville, USA; 2 Emergency Medicine, Campbell University School of Osteopathic Medicine, Lillington, USA; 3 Critical Care Medicine, Cape Fear Valley Medical Center, Fayetteville, USA; 4 Cardiology, Cape Fear Valley Medical Center, Fayetteville, USA; 5 Cardiovascular Medicine, Creighton University School of Medicine, Omaha, USA

**Keywords:** drug and substance abuse, janeway lesions, osler nodes, intracranial hemorrhage (ich), blood culture-negative endocarditis

## Abstract

Infectious endocarditis (IE) is a diagnosis in which thorough evaluation must be performed and certain diagnostic criteria must be met. Thorough history and detailed physical examination can affect and guide the management of a patient from the very beginning. One of the main causes of endocarditis that physicians deal with in the hospital is intravenous drug abuse. This case report is of a 29-year-old male presenting to a rural emergency department with a two-week history of altered mental status after being struck on the head with a metal pipe. The patient also endorsed using intravenous drugs along with subcutaneous injections (skin popping). The patient was initially treated as a traumatic intracranial hemorrhage, but it was later found to be secondary to septic emboli from blood culture-negative endocarditis. Throughout this case report, we will approach the difficulties of diagnosing IE in a patient who represented many of the less common findings including dermatologic manifestations of diseases such as Osler nodes and Janeway lesions.

## Introduction

An infection that enters the bloodstream, travels to the heart, and infiltrates coronary tissue is what causes infectious endocarditis (IE); with the most frequent cause of the disease being *Staphylococcus aureus* [1]. Around 5% of the general population is at risk for developing IE, making it a prevalent illness [2]. Any form of dental work, a urinary tract infection, a lung infection, a gastrointestinal infection, a skin condition, an intravenous drug addiction problem, surgery, and intravenous cannulation are all potential entry points [2]. Infection with Gram-positive Streptococci, Staphylococci, and Enterococci is the predominant cause of IE [3]. Collectively, these three categories account for 80-90% of all cases, with *Staphylococcus aureus*, in particular, being the culprit in about 30% of cases in the industrialized world [3]. It is possible to detect the classic immunologic and hemorrhagic sequelae of IE on dermatologic examination [4]. Less than 10% of all cases are characterized by painless Janeway lesions or Osler nodes (painful subcutaneous nodules typically occurring on the palms and the soles) [4]. Here we are presenting an interesting case of a patient who used the skin-popping technique to inject drugs into his body. He initially presented at an outside hospital for confusion after being hit with a metal object a few months prior, and their conclusion was his cerebral hemorrhage was from the traumatic impact. He also was found to have multiple subcutaneous erythematous lesions that were thought to be related to his skin-popping. This report will cover the signs and symptoms to look for in a physical examination, and how the clinical picture may not be so straightforward.

## Case presentation

**Figure 1:**
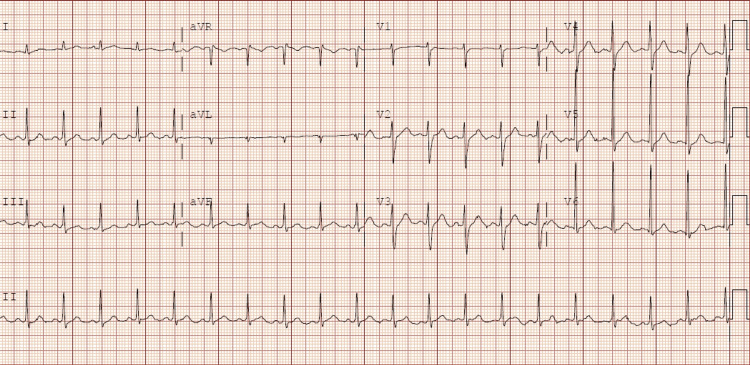
Electrocardiogram that showed sinus tachycardia with a rate of 119 beats per minute.

**Figure 2:**
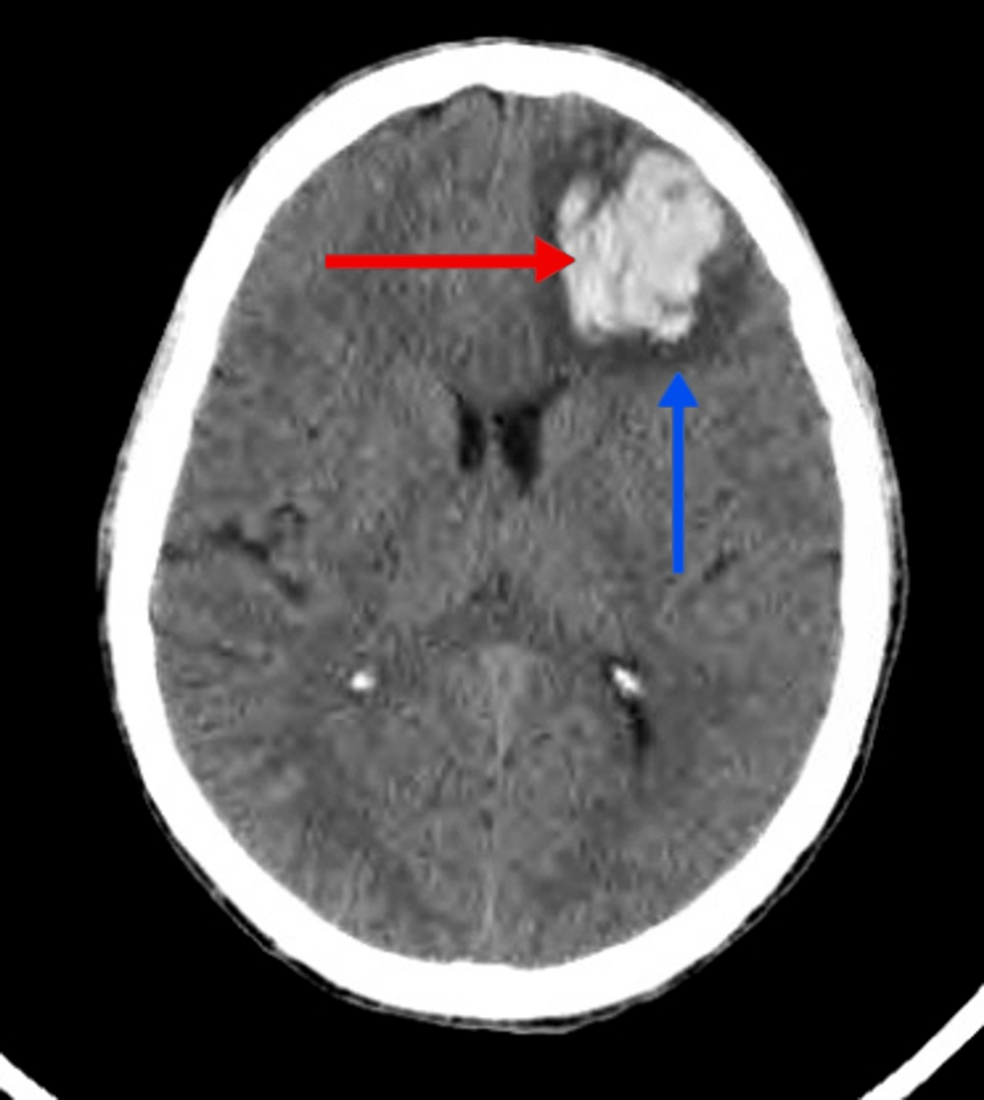
A computerized tomography (CT) scan of the head showing a large hemorrhagic focus throughout the left frontal lobe (red arrow) with a large amount of surrounding edema (blue arrow) creating mass effect upon the adjacent frontal horn of the left lateral ventricle.

A 29-year-old male with a past medical history of tobacco use and intravenous drug use was transferred from an outside facility for traumatic hemorrhage of the left frontal lobe after being struck on the head by a metal pipe. The history was obtained from the girlfriend at bedside, and she reported that the patient was struck on the head two months before initially presenting to the outside facility. He was acting normal after being struck on the head, and he only became confused over the past 24 hours prior to arrival along with fevers up to 104 degrees Fahrenheit. Furthermore, the patient’s girlfriend stated that the patient had increased his heroin by skin popping, and marijuana and Roxicodone use in the previous two weeks. She said that he was no longer able to find any veins on himself, so he started skin-popping his heroin. The patient had been complaining of painful lesions located all over his body but would not allow anyone to look at or touch the lesions due to pain. The patient thought it was secondary to his reusing dirty needles. He was intubated at the other facility for airway protection because he was unresponsive when emergency medical services brought him into the emergency department. It was reported by the outside facility that the patient had innumerable superficial areas of infection from skin popping. Blood work can be seen in Table 1. The initial electrocardiogram (Figure 1) showed sinus tachycardia with a rate of 119 beats per minute. A computed tomography (CT) scan of the head without contrast showed a large hemorrhagic focus throughout the left frontal lobe with a large amount of surrounding edema creating a mass effect upon the adjacent frontal horn of the left lateral ventricle (Figure 2). The impression of this CT suggested acute hemorrhage versus embolic phenomena versus mycotic aneurysm.

**Table 1:** Blood work that was obtained initially and throughout hospital course.

Laboratory Test	Patient's Laboratory Values	Reference Range
Complete Blood Count		
White Blood Cell Count	16.3 x10*3/uL	4.5 - 12.5 x10*3/uL
Hemoglobin	12.8 g/dL	12.0 - 16.0 g/dL
Mean Corpuscular Volume	88.9 fL	81.0 - 99.0 fL
Platelets	55 x10*3/uL	150 - 450 x10*3/uL
Comprehensive Metabolic Panel		
Sodium	132 mmol/L	136 - 145 mmol/L
Potassium	3.9 mmol/L	3.5 - 5.1 mmol/L
Bicarbonate	29 mmol/L	21 -32 mmol/L
Chloride	93 mmol/L	98 - 107 mmol/L
Blood Urea Nitrogen	31 mg/dL	7 - 25mg/dL
Creatinine	1.02 mg/dL	0.60 mg/dL
Glucose	139 mg/dL	74 - 106 mg/dL
Aspartate aminotransferase	40 U/L	15 - 37 U/L
Alanine transaminase	23 U/L	12 - 78 U/L
Glomerular filtration rate	>60.0 mL/min/1.73m*2	>60.0 mL/min/1.73m*2
Albumin	2.0 g/dL	3.5 - 5.7 g/dL
Magnesium	2.5 mg/dL	1.9 - 2.7 mg/dL
Arterial Blood Gas		
Arterial pH	7.50 pH	7.35 - 7.45 pH
Oxygen	85 mmHg	75 - 100 mmHg
Carbon Dioxide	34 mm Hg	35.0 - 45.0 mm Hg
Bicarbonate on Arterial Blood Gas	26.5 mEq/L	22.0 - 26.0 mEq/L
Other Blood Tests		
Ammonia	17 umol/L	11 - 32 umol/L
Thyroid Stimulating Hormone	2.412 uIU/mL	0.358 - 3.740 uIU/mL
Free T4	1.07 ng/dL	0.76 - 1.46 ng/dL
Ethanol Level	<3 mg/dL	<3 mg/dL
High Sensitivity Troponin	10 pg/mL	2 -20 pg/mL
Three-Hour High Sensitivity Troponin	11 pg/mL	2 -20 pg/mL
B-Type Natriuretic Peptide	<100 pg/mL	< 100 pg/mL
Rapid Plasma Reagin	Negative	Negative
Q Fever Antibodies, IgG	Negative: <1:16	Negative: <1:16
*Mycoplasma pneumoniae* Antibody, IgM	< 770 U/mL	0 - 769 U/mL
*Brucella* Antibody IgM	Negative	Negative
*Chlamydia pneumoniae* IgM	Negative: <1:10	Negative: <1:10
Fungitell® Assay	<31 pg/mL	<80 pg/mL
*Bartonella* DNA Polymerase Chain Reaction	Negative	Negative
Human Immunodeficiency Virus screen 4th Generation with Reflex	Non-Reactive	Non- Reactive
Lactic Acid	1.3 mmol/L	0.5 - 2.0 mmol/L
Urine Drug Screen		
Benzodiazepine	Negative, None detected	Negative, None detected
Cocaine	Negative, None detected	Negative, None detected
Tetrahydrocannabinol	Negative, None detected	Negative, None detected
Phencyclidine	Negative, None detected	Negative, None detected
Amphetamine	Negative, None detected	Negative, None detected
Opiate	Negative, None detected	Negative, None detected
Blood Culture 1	Negative, No Growth	Negative, No Growth
Blood Culture 2	Negative, No Growth	Negative, No Growth

**Figure 3:**
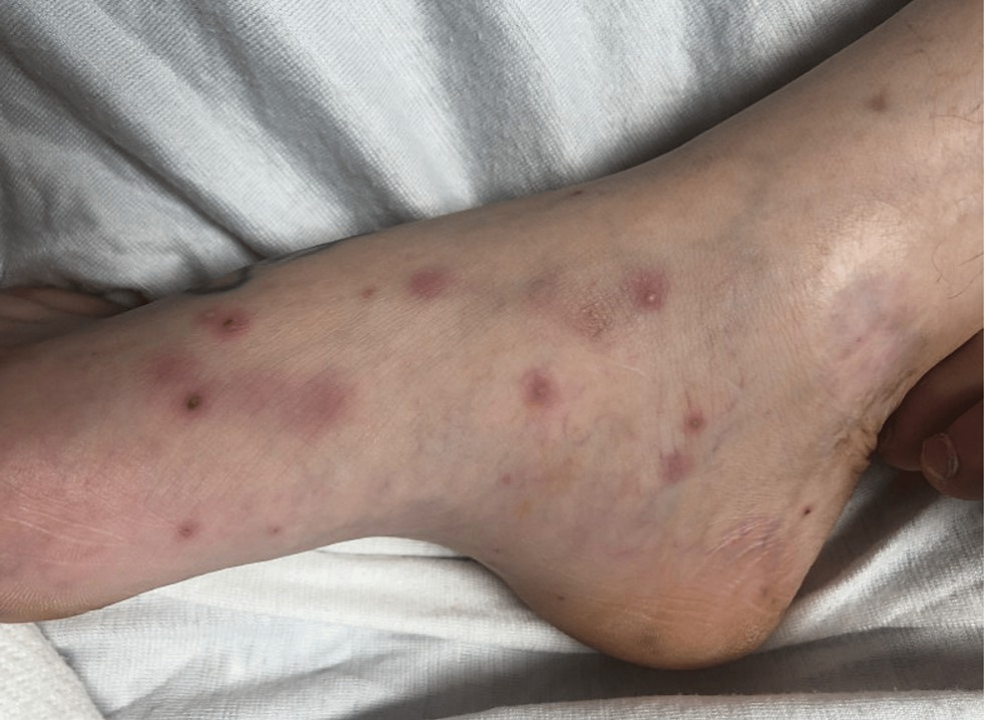
Osler nodes seen as dark, raised, and purplish covering the medial aspect of the patient's right foot.

**Figure 4:**
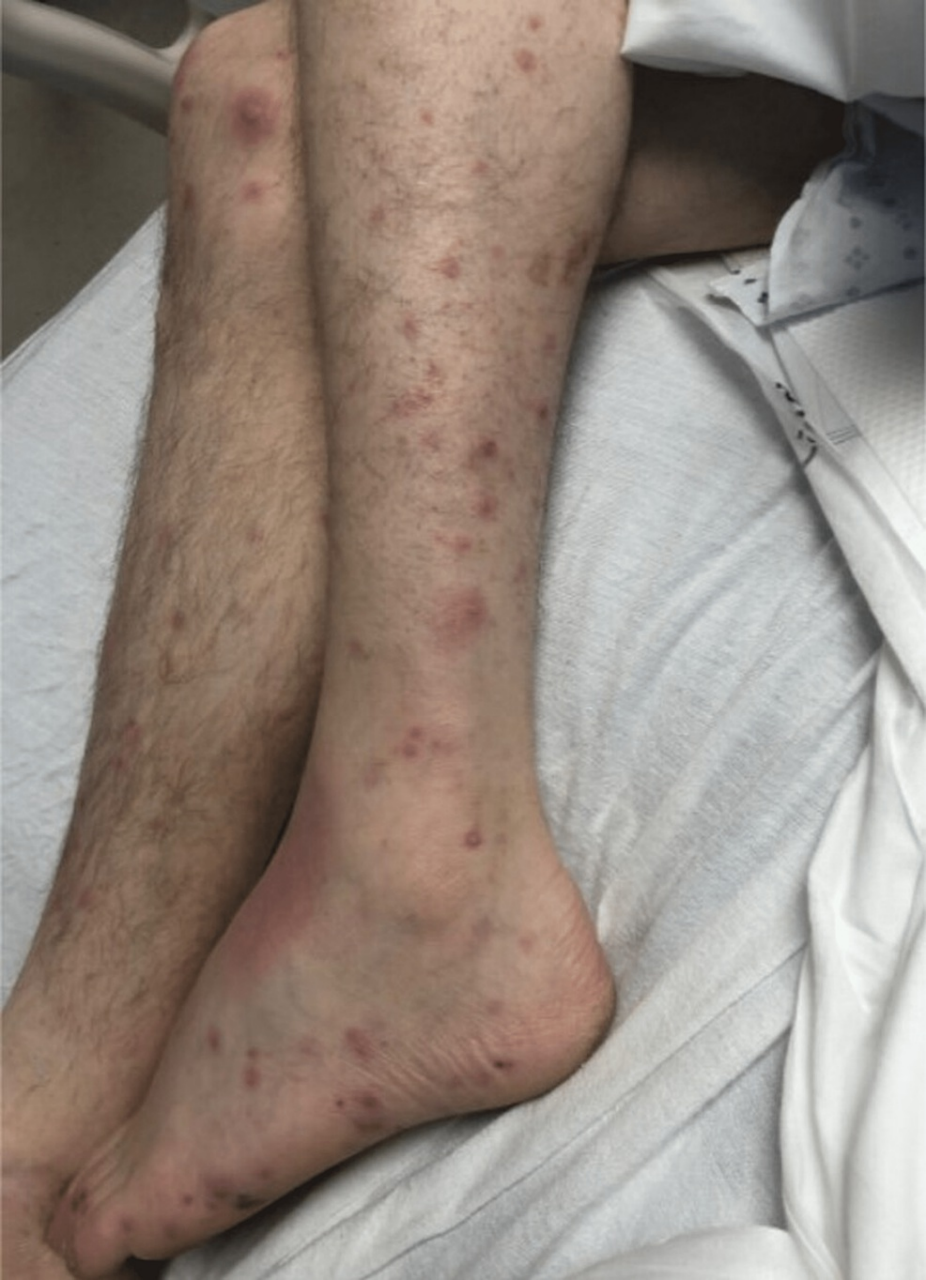
Osler nodes seen over the anterior and medial aspect of the patent bilateral lower extremities.

**Figure 5:**
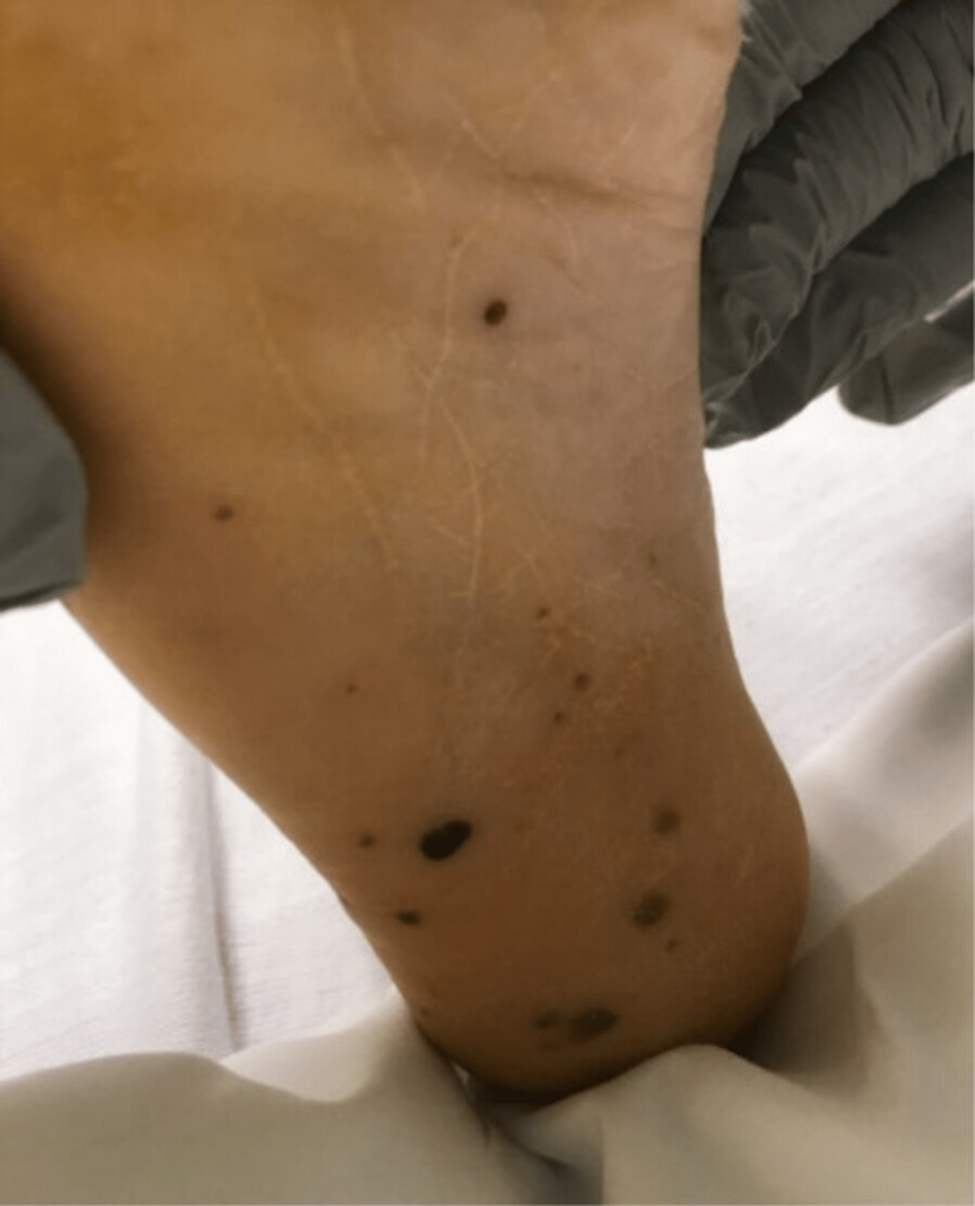
Janeway lesions seen on the sole of the patient's right foot.

**Figure 6:**
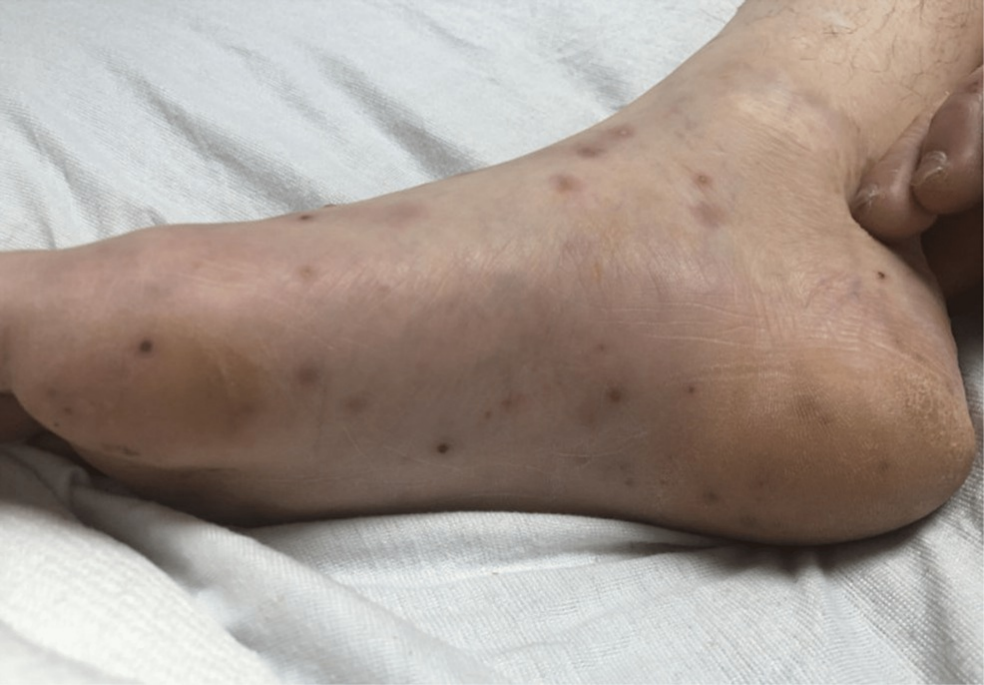
Janeway lesions seen on the sole of the right foot with Osler nodes seen on the medial aspect of the right foot and ankle.

**Figure 7:**
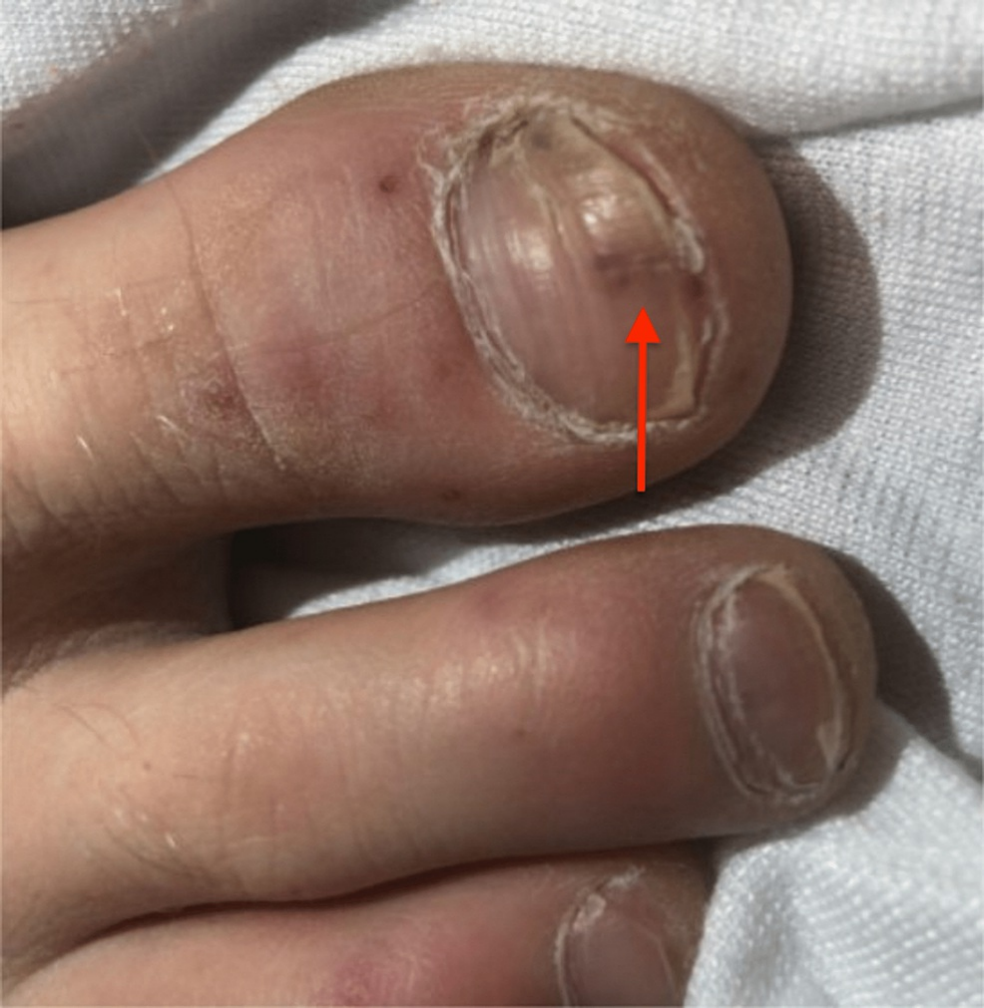
Splinter hemorrhage seen at the tip of the red arrow on the first digit of the right foot.

**Figure 8:**
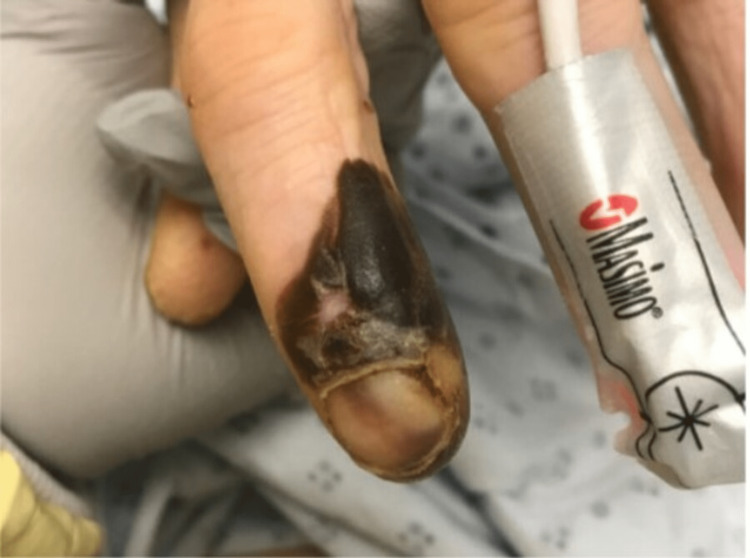
Necrosis of the distal aspect of the right-hand second digit.

**Figure 9:**
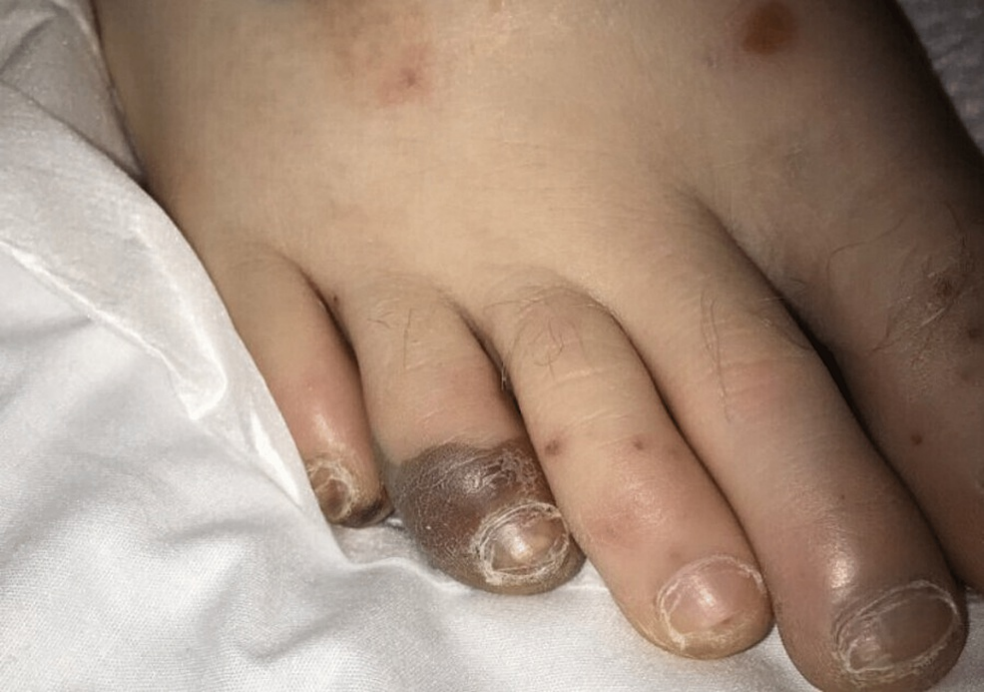
Necrosis of the fourth digit of the right foot and the tip of the fifth digit.

The patient transfer request was initiated and he was transferred to the intensive care unit. When he arrived, the patient underwent a detailed and meticulous physical examination. The physical examination findings included diffuse areas of erythematous nodules over the patient's bilateral upper and lower extremities suggestive of Osler nodes (Figures 3, 4) and Janeway lesions (Figures 5, 6) as well as splinter hemorrhages (Figure 7). The patient also had necrotic appearing digits involving the right second finger (Figure 8), right fourth toe (Figure 9), and the tip of the right fifth toe (Figure 9). A CT head and neck angiogram showed widely patent vessels and no aneurysm or stenosis. A transthoracic echocardiogram (TTE) results demonstrated moderate mitral valve regurgitation and a non-mobile and small vegetation on the posterior leaflet of the mitral valve measuring 10mm x 6mm (Videos 1, 2). A request was placed for Neurosurgery to evaluate, and they recommended no surgical intervention at this time. Cardiothoracic surgery was also asked to come and evaluate, and they reported no surgical intervention at this time. Neurosurgery and Cardiothoracic surgery believed the hemorrhage appeared to be thromboembolic in nature from the patient's endocarditis. The patient’s IV antibiotics were escalated to include IV cefepime and vancomycin, per infectious disease. The patient’s blood cultures were continuously negative; however, the patient’s leukocytosis remained elevated. Blood culture-negative endocarditis (BCNE) labs were drawn including *Coxiella burnetii* (Q fever), *Bartonella* spp, *Brucella* spp, *Chlamydia pneumoniae*, and *Mycobacteria* spp, which all resulted as negative. The patient was eventually extubated and transferred out of the intensive care unit. After approximately 12 days, he was discharged from the hospital and was ultimately lost to follow-up. 

**Video 1:** Transthoracic echocardiogram demonstrating ejection fraction greater than 55%, moderate mitral valve regurgitation, non-mobile and small vegetation on the posterior leaflet of the mitral valve measuring 10mm by 6mm.

**Video 2:** Transthoracic echocardiogram demonstrating ejection fraction greater than 55%, non-mobile and small vegetation on the posterior leaflet of the mitral valve measuring 10mm by 6mm.

## Discussion

IE is a relatively uncommon disorder that occurs 3-10 times per 100,000 annually [5]. A hospital mortality rate of 22%, rising to 40% after five years, reflects the poor prognosis despite diagnostic and therapeutic advances [5]. When no causal microbe can be cultured using standard blood culture techniques, it is known as blood culture-negative infective endocarditis (BCNIE) [6]. Five to ten percent of all cases of endocarditis are caused by BCNIE [6]. According to an 820-case European investigation, 20% of confirmed IE patients had negative blood cultures [6]. 

IE complications can be vast and vary on factors such as the pathogen that causes the infection, the length of the illness before therapy, and the type of therapy. In 22-50% of instances, there is systemic embolization; emboli can impact major arteries, mostly affecting the central nervous system but also other organs [7]. Neurological complications develop in 20-40% of patients with IE and represent a dangerous subset of complications [7]. IE is known to cause intracranial hemorrhage (ICH). Intraparenchymal hemorrhage, which appears to be caused by a burst mycotic aneurysm, is the most frequent cerebral hemorrhagic complication of endocarditis [8]. 

IE is a diagnosis in which thorough evaluation must be performed and certain diagnostic criteria must be met. IE can be diagnosed via the Modified Duke criteria, which includes major and minor criteria [3]. Major criteria for diagnosis include positive blood cultures and evidence of endocardial involvement; while minor criteria for diagnosis include a predisposition for the disease, the presence of fever, vascular phenomena, immunologic phenomena, and microbiologic evidence [3]. To diagnose using the modified Duke criteria a patient will need to have one of the following: 1) two major criteria, 2) one major and two minor criteria, or 3) five minor criteria [9]. 

Skin popping is a technique for injecting illegal drugs into the subcutaneous skin [10]. This is done in order to achieve slower absorption, lower danger of overdose, and simpler administration compared to intravenous drug use [10]. This type of injection involves injecting illicit chemicals, particularly cocaine, opiates, and barbiturates into the skin [10]. Bacterial infections, including abscesses and cellulitis, are the most frequent acute cutaneous side effects of skin bursting [11]. According to a study, skin-popping drug users had roughly five times the risk of developing an abscess or cellulitis as those who used intravenous injection [9]. Most frequently, antibiotic treatment targeted at *Staphylococcus aureus* and *Streptococcus* spp. cures uncomplicated cellulitis caused by skin popping [11]. This case presented an unusual complication of skin popping, as he developed left-sided endocarditis. 

## Conclusions

The patient was ultimately diagnosed with IE as he met one major and more than three minor criteria including the major criteria of evidence of endocardial involvement and the minor criteria of predisposition including intravenous drug use, fever of up to 104 degrees Fahrenheit, vascular phenomena including intracranial hemorrhage as well as Janeway lesions and immunologic phenomena of Osler nodes. This case report illustrates the importance of a detailed and meticulous history and physical examination. IE is not always a straightforward diagnosis, especially when confounding factors are present as seen in our patient. From the beginning, it was assumed that the patient's intracranial hemorrhage was induced by the traumatic impact of the metal pipe. All of his symptoms were a result of endocarditis from skin popping his heroin. This is an interesting case as our patient ultimately had left-sided BCNE secondary to skin popping. 

## References

[1] Munshi R, Pellegrini JR, Tsiyer AR, Barber M, Hai O (2021). "To fix a broken heart": an unusual case of infective endocarditis involving the mitral valve with perforation and hemodynamic instability. Cureus.

[2] Houghton T, Kaye GC, Meigh RE (2002). An unusual case of infective endocarditis. Postgrad Med J.

[3] Yallowitz A, Decker L (2022). Infectious endocarditis. StatPearls [Internet].

[4] Murdoch DR, Corey GR, Hoen B (2009). Clinical presentation, etiology, and outcome of infective endocarditis in the 21st century: the International Collaboration on Endocarditis-Prospective Cohort Study. Arch Intern Med.

[5] Khan O, Shafi AM, Timmis A (2016). International guideline changes and the incidence of infective endocarditis: a systematic review. Open Heart.

[6] Ebato M (2018). Blood culture-negative endocarditis. Advanced Concepts in Endocarditis.

[7] Mocchegiani R, Nataloni M (2009). Complications of infective endocarditis. Cardiovasc Hematol Disord Drug Targets.

[8] Khoury J, Cho S, Rice C (2018). Intracranial hemorrhage in infective endocarditis: underlying arterial and parenchymal disease. Stroke.

[9] Topan A, Carstina D, Slavcovici A, Rancea R, Capalneanu R, Lupse M (2015). Assesment of the Duke criteria for the diagnosis of infective endocarditis after twenty-years. An analysis of 241 cases. Clujul Med.

[10] Saporito RC, Lopez Pineiro MA, Migden MR, Silapunt S (2018). Recognizing skin popping scars: a complication of illicit drug use. Cureus.

[11] Ebright JR, Pieper B (2002). Skin and soft tissue infections in injection drug users. Infect Dis Clin North Am.

